# Identifying predictors of patient radiation dose during uterine artery embolisation

**DOI:** 10.1002/jmrs.450

**Published:** 2020-11-13

**Authors:** Don J. Nocum, John Robinson, Mark Halaki, Eisen Liang, Nadine Thompson, Michelle Moscova, Warren Reed

**Affiliations:** ^1^ San Radiology & Nuclear Medicine Sydney Adventist Hospital Wahroonga New South Wales Australia; ^2^ Discipline of Medical Imaging Science School of Health Sciences, Faculty of Medicine and Health The University of Sydney New South Wales Australia; ^3^ Discipline of Exercise and Sports Science School of Health Sciences, Faculty of Medicine and Health The University of Sydney New South Wales Australia; ^4^ Department of Radiology Sydney Adventist Hospital Clinical School University of Sydney Wahroonga New South Wales Australia; ^5^ Faculty of Medicine School of Medical Sciences University of New South Wales New South Wales Australia

**Keywords:** dose optimisation, interventional radiology, radiation dose, regression analysis, uterine artery embolisation

## Abstract

**Introduction:**

Uterine artery embolisation (UAE) is regarded as a safe and effective treatment for symptomatic uterine fibroids and/or adenomyosis. Dose reduction during UAE is critical for this reproductive‐age patient population to minimise the risks of radiation‐induced effects. The aim of this study was to identify the predictors of radiation dose which can be controlled and optimised for patients during UAE.

**Methods:**

A total of 150 patients between June 2018 and August 2019 were included in this study. Demographic and clinical information such as age, body mass index (BMI), total number of fibroids, total fibroid volume, total uterus volume and dosimetric measurements on Dose Area Product (DAP), Air Kerma (AK) and fluoroscopy time were recorded. Total digital subtraction angiography (DSA), total conventional roadmap (CRM), total last‐image hold (LIH) and total fluoroscopy were calculated from the dose report. Multiple linear regression analysis was used to identify the independent predictor variables of total dose (DAP) using a regression model.

**Results:**

Total DSA, total CRM and total LIH were identified as the determinants of dose for UAE (*P* < 0.05) and together accounted for 95.2% of the variance.

**Conclusions:**

This study identified the key imaging predictors of dose for UAE. Total DSA, total CRM and total LIH were shown to have a greater impact on the outcome DAP compared to other demographic or dosimetric measurements. Optimisation of these predictors during future UAE procedures can facilitate radiation dose reduction to the pelvis and reproductive organs.

## Graphical abstract

The predictors of patient radiation dose have been identified for the interventional radiology procedure known as uterine artery embolisation (UAE). A regression model entered and identified total digital subtraction angiography (DSA), total conventional roadmap (CRM) and total last‐image hold (LIH) as the determinants of dose over demographic and other angiographic variables. Control of these dose predictors can potentially minimise radiation dose exposure to this reproductive‐age patient population. Dose optimisation can be attained to reduce the risks of tissue effects and/or stochastic effects associated with irradiating the pelvis region.

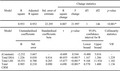

## Introduction

Uterine artery embolisation (UAE) is a uterine‐sparing, minimally invasive procedure that can be undertaken as a surgical alternative for treating women with symptomatic fibroids and/or adenomyosis.^1^, ^2^, ^3^ Angiographic imaging during UAE exposes the female pelvic area to varying levels of ionising radiation.^4^ Hence, the clinical knowledge of key predictors of dose can further monitor and optimise patient radiation dose during this procedure. Radiology prediction models in previous studies have been developed to include multiple variables that can objectively predict the radiation dose for a specific procedure.^5^, ^6^, ^7^, ^8^ The regression model in this study, however, facilitates the identification of the entered dose predictor variables and not the predicted dose outcome. Implementation of this model can assist interventional radiologists and radiographers to perform intra‐procedural dose optimisation and subsequent reduction of cumulative Dose Area Product (DAP) and AK values. For UAE patients, this can reduce the risks of any potential tissue effects and/or stochastic effects for this mainly reproductive‐age population. The variables entered and identified could also potentially be extrapolated to other similar interventional radiology procedures for dose optimisation.

Medical imaging, which involves ionising radiation, has inherent radiation‐induced side‐effects that can be minimised by practising radiation safety. The International Commission on Radiological Protection (ICRP) reported in 2007 that females are 1.6 times more radiosensitive to radiation than males and therefore advised minimal exposure to radiosensitive reproductive organs such as the uterus and ovaries.^9^ Nikolic et al^10^ stated that the skin entrance dose for UAE procedures should not exceed the threshold of 2 Gy to prevent any radiation‐induced injury such as skin erythema and epilation.^11^, ^12^, ^13^, ^14^ Goodman & Amurao suggested that ensuring exposures are kept as low as reasonably achievable (ALARA) can reduce the possibility of a stochastic effect from occurring.^15^ There is a paucity of literature on patient radiation dose during UAE, particularly in the Australian context. There are currently no known regression models that can be used to guide interventional radiologists and radiographers to control specific dose predictors in order to minimise female patient radiation dose, thus identifying these determinants has direct implications on improving radiation dose practices.

This study was conducted at a specialist interventional radiology centre which performs a high volume of UAE procedures. This is also the first known investigation on baseline radiation dose exposure data on women undergoing UAE in Australia. The outcomes of this study can be used in a continuous quality improvement (CQI) programme to provide solutions for dose management and improve patient care through dose optimisation strategies. The purpose of this study was to identify the key determinants of radiation dose for UAE patients which can be controlled and optimised in future UAE procedures.

## Methods

### Ethics approval and participant informed consent

This study was reviewed and approved by the Sydney Adventist Hospital Human Research Ethics Committee. All patient records and data relating to this study were anonymised and de‐identified.

### Study population

In this prospective study, a total of 150 patients underwent a UAE procedure between June 2018 and August 2019. The inclusion criteria were as follows: (1) first UAE for the treatment of symptomatic uterine fibroids and/or adenomyosis, (2) successful completion of embolisation of the left uterine artery (LUA) and right uterine artery (RUA) via a transfemoral approach, and (3) use of a non‐modified, standard departmental protocol for UAE under angiographic imaging. Patients with a previous UAE or prior gynaecological pelvic surgery were excluded. Radiation dose data on a separate group of 12 patients from four different BMI groups were used to validate the preliminary regression model. The BMI < 18.5 kg/m^2^, 18.5 ≤ BMI ≤24.9 kg/m^2^, 25 ≤ BMI ≤29.9 kg/m^2^ and BMI ≥ 30 kg/m^2^ groups were considered underweight, normal weight, overweight and obese, respectively.^16^

### Data collection

The participants had undergone a pre‐procedural magnetic resonance imaging (MRI) scan to verify the diagnosis of the uterine fibroids (including the number and size) and/or adenomyosis (based on the junctional zone thickness). The interventional radiologist had performed a clinical assessment prior to the procedure and all patients were considered suitable for UAE. As the baseline for this study, the following demographic and clinical information was obtained and recorded: age (years), height (cm), body mass (kg), total number of fibroids (*n*), total fibroid volume (cm^3^), total uterus volume (cm^3^) and body mass index/BMI (kg/m^2^). Post‐procedure the following data on radiation dose parameters was collected from the dose report: DAP (Gy·cm^2^), AK (Gy) and fluoroscopy time (min). Dosimetric data on DAP for the imaging modes used during each stage of the procedure were recorded. These imaging modes included the following: (1) digital subtraction angiography (DSA), (2) conventional roadmap (CRM) or navigate (Philips Healthcare 2019) (3) last‐image hold (LIH) and (iv) fluoroscopy. Radiation doses were measured by a calibrated DAP meter (DIAMENTOR, PTW; Freiburg, Germany) fitted on the exit surface of the collimator assembly. AK values were computationally estimated at the interventional reference point (IRP).

### UAE imaging modes

A detailed UAE protocol for this centre has previously been described by Liang et al.^1^ All UAE procedures were performed on a flat‐detector angiography unit (Philips Allura Xper FD20; Philips Healthcare, Netherlands). The four imaging modes: DSA, CRM, LIH and fluoroscopy were available for use at the discretion of the interventional radiologist and guidance by the radiographer to visualise and manipulate catheters through the vasculature.

DSA utilises pulsed mode image acquisition, where a high tube current X‐ray pulsed image is collected at the rate of a few frames per second (fps) during injection of contrast material and subtracted from a stored mask obtained prior to the injection.^17^, ^18^ A single frame or combined frames from a DSA run can be used as a roadmap. Due to the relatively higher dose of DSA, this imaging mode produces a high clarity roadmap for use during live fluoroscopy and device manipulation. CRM involves a continuous pulsed fluoroscopic beam that is delivered at a fixed rate of 15 pulses per second (pps) on this system. This is a single peak opacified image with high vessel contrast and is the next favourable imaging mode used for a roadmap. LIH is achieved by interposing various filters into the x‐ray beam inside the tube housing, where the fluoroscopy captured images can be used as a roadmap.^17^ A single or combined frames from a LIH can be also be used as a roadmap. However, due to the lower dose acquired during fluoroscopy, the image has less clarity and is dependent on the amount of contrast medium injected. Post‐processing of the contrast, brightness and edge enhancement can improve the clarity of this particular roadmap.

The use of DSA for the aortogram, LUA and RUA and any ovarian artery supply were acquired at a multi‐phase acquisition pulsed rate setting of 3 fps for 3 s, 2 fps for 2 s and 1 fps. The fluoroscopy pulsed frequency at the low‐dose fluoro setting was 7.5 fps, and the medium‐dose and high‐dose fluoro settings were at 15 fps. The selected fluoroscopy pre‐filters were set at 0.90 mm Cu and 1.00 mm Al. The total DSA, total CRM, total LIH and total fluoroscopy radiation doses were calculated by the angiography unit based on automated tube potential (kVp) and tube current (mAs) values and dependent on the frames per second, exposure duration and size of the patient.

### Statistics

This study involves a multivariable analysis of the independent variables found with UAE procedures that are contributing to the radiation dose‐dependent variable, that is DAP. Data were analysed using SPSS Statistics version 25 (IBM Corporation, 2017). To evaluate the associations between DAP and each of the nine predictor variables, BMI, total number of fibroids, total fibroid volume, total uterus volume, fluoroscopy time, total DSA, total CRM, total LIH and total fluoroscopy Pearson’s and Spearman’s rho correlation coefficients were used as not all variables were normally distributed. Based on Cohen’s recommendation^19^, the following criteria were used to evaluate the correlation coefficients: nil (*r* < 0.2), weak (0.2 ≤ *r* < 0.5), moderate (0.5 ≤ *r* < 0.8) and strong (*r* ≥ 0.8).

Dose Area Product was the most reliable predictor outcome variable applied to the model. To identify the predictors of dose for a UAE procedure, a multiple linear regression analysis with stepwise elimination was performed using the following predictor variables: BMI, total number of fibroids, total fibroid volume, total uterus volume, fluoroscopy time, total DSA, total CRM and total LIH. Scatter plots of the predictor variables and DAP were visually checked to confirm the assumption of a linear association between the predictor variables and DAP. Total fluoroscopy was excluded due to the multi‐collinearity with the other predictor variables. The criteria used for the stepwise selection process was based on *P*‐values. A *P*‐value < 0.05 was considered statistically significant.

## Results

Table 1 shows the patient demographic and clinical information for the 150 patients in this study. The median age was 45 years, and the median BMI was 24 kg/m^2^. The median total uterus volume was 233 cm^3^. There were 120 out of 150 patients with symptomatic fibroids and the reported median total fibroid volume was 176 cm^3^. For these patients, the median total number of fibroids was two. The radiation dose parameters and radiation dose measurements from each imaging mode are provided in Table 2. The median DAP was 113.1 Gy·cm^2^ and the median AK was 0.5 Gy. The median fluoroscopy time was 11.1 minutes. For the imaging modes, total DSA contributed the highest dose with a median DAP of 75.3 Gy·cm^2^. This was followed by total fluoroscopy, total CRM and total LIH with median values of 19.8, 12.5 and 0.7 Gy·cm^2^, respectively.

**Table 1:** Demographic and clinical information for all UAE patients.

Demographic and clinical information	*n*	Median	IQR	Range
Age (years)	150	45	6	28–65
Height (cm)	150	163	8	146–183
Body mass (kg)	150	65	18	45–120
BMI (kg/m^2^)	150	24	6	18–36
Total number of fibroids (*n*)	120^†^	2	2	1–10
Total fibroid volume (cm^3^)	120^†^	176	249	2–1358
Total uterus volume (cm^3^)	150	233	233	40–2577

Note:

This table demonstrates the descriptive statistics for the demographic and clinical information for the 150 UAE patients included in this study. The total number of fibroids, total fibroid volume and total uterus volume values were recorded from the pre‐procedural MRI report. (UAE = uterine artery embolisation, IQR = interquartile range, BMI = body mass index).

^†^ A total of 120 patients presented with symptomatic fibroids (the remaining 30 patients had symptomatic adenomyosis); IQR – Interquartile Range

**Table 2:** Radiation dose parameters for all UAE patients.

Radiation dose parameter	*n*	Median	IQR	Range
DAP (Gy·cm^2^)	150	113.1	96.5	(21.9 – 792.8)
AK (Gy)	150	0.5	0.5	(0.1 – 2.4)
Fluoroscopy time (min)	150	11.1	6.8	(6.2 – 33.6)
Total DSA (Gy·cm^2^)	150	75.3	67.5	(7.7 – 543.6)
Total CRM (Gy·cm^2^)	150	12.5	10.2	(0 – 61.8)
Total LIH (Gy·cm^2^)	150	0.7	1	(0.1 – 21.7)
Total fluoroscopy (Gy·cm^2^)	150	19.8	25	(3.9 – 372.7)

Note:

This table demonstrates the descriptive statistics for the radiation dosimetric parameters for all 150 UAE patients included in this study. All measurements are in SI or SI‐derived units. (UAE = uterine artery embolisation, DAP = Dose Area Product, AK = Air Kerma, IQR = interquartile range, DSA = digital subtraction angiography, CRM = conventional roadmap, LIH = last‐image hold).

Table 3 shows the Pearson’s correlation coefficient and Spearman’s rho values between DAP and each of the nine predictor variables. All variables were significant (*P* < 0.05) except for total fibroid volume and total uterus volume.

**Table 3:** Correlation between DAP and all nine predictor variables

	*n*	Spearman’s rho		Pearson’s	
		Correlation Coefficient	*P*‐value (2‐tailed)	Correlation Coefficient	*P*‐value (2‐tailed)
BMI	150	0.59	<0.001*	0.45	<0.001*
Total number of fibroids	120^†^	0.29	0.001*	0.23	0.012*
Total fibroid volume	120^†^	0.08	0.404	0.44	<0.001*
Total uterus volume	150	0.12	0.196	0.49	<0.001*
Fluoroscopy time	150	0.39	<0.001*	0.39	<0.001*
Total DSA	150	0.94	<0.001*	0.93	<0.001*
Total CRM	150	0.59	<0.001*	0.43	<0.001*
Total LIH	150	0.49	<0.001*	0.54	<0.001*
Total fluoroscopy	150	0.82	<0.001*	0.82	<0.001*

Note:

Pearson’s and Spearman’s rho correlations coefficients between DAP and all nine predictor variables. All statistically significant *P*‐values were denoted with an (*) symbol. (DAP = Dose Area Product, BMI = body mass index, DSA = digital subtraction angiography, CRM = conventional roadmap, LIH = last‐image hold).

* *P*‐value < 0.05 is statistically significant.

^†^ 120 patients with symptomatic fibroids.

The multiple linear regression revealed that total DSA, total CRM and total LIH were significant (*P* < 0.05) predictors of DAP and in total accounted for 95.2% of the variance (Table 4). The analysis demonstrated that 86.9% of the variance was accounted for by total DSA, a further 7.6% was accounted for when adding total CRM and a further 0.7% was accounted for when adding total LIH to the regression model. During the stepwise process, the following factors were excluded: BMI, total number of fibroids, total fibroid volume, total uterus volume and fluoroscopy time. The inclusion of total fibroid volume to this model only accounted for an additional 0.1% of the variance. Total fluoroscopy showed multi‐collinearity with several of the other predictor variables and was therefore not included in the final analysis. There was no correlation between total fibroid volume and fluoroscopy time (*r* = 0.02). BMI and total number of fibroids were significant on a bivariate level but were not considered as predictor variables as they did not reach significance in the multivariable model.

**Table 4:** Multiple linear regression model summary and coefficients^a^ for the predictor variables

Model	R square	Adjusted R square	Std. error of estimate	R square change	Change statistics			*P*‐value
					F change	df1	df2	
	0.953	0.952	22.295	0.007	21.997	1	146	<0.001*

Model	Unstandardised coefficients		Standardised coefficients beta	t	*P*‐value	95.0% confidence interval for B		Collinearity statistics
	B	Std. error				Lower bound	Upper bound	VIF
(Constant)	−2.232	3.667	‐	−0.609	0.544	−9.480	5.016	‐
Total DSA	1.226	0.030	0.816	40.855	<0.001*	1.167	1.286	1.229
Total LIH	10.551	0.700	0.285	15.077	<0.001*	9.168	11.934	1.102
Total CRM	0.985	0.210	0.090	4.690	<0.001*	0.570	1.400	1.136

Note:

This table demonstrates the multiple linear regression analysis performed on DAP and all nine predictor variables. The top section of this table relates to the all‐potential‐predictors model. All statistically significant *P*‐values were denoted with an (*) symbol. (DAP = Dose Area Product, DSA = digital subtraction angiography, CRM = conventional roadmap, LIH = last‐image hold).

^a^ DAP = Dependent Variable.

* *P*‐value < 0.05 is statistically significant;

The regression model that entered the identified dose predictor variables for patients undergoing UAE was found to be:

$$DAP=1.226\left(total\;DSA\right)+10.551\left(total\;LIH\right)+0.985\left(total\;CRM\right),$$

where total DSA, total LIH and total CRM were measured in Gy·cm^2^.

For the preliminary validation of this model to test its potential as a prediction model to estimate dose (DAP) in future studies, radiation dose data on 12 UAE patients from four different BMI groups were substituted into the regression equation. These patients were separate to the 150 patient cohort. Table 5 shows the predicted DAP and actual DAP for these 12 UAE patients (*a*‐*l*), following the use of actual total DSA, total CRM and total LIH radiation dose quantities. This table demonstrates that the regression equation has a high predictive capacity of 91.1‐99.3%.

**Table 5:** Preliminary validation of the regression equation for 12 UAE patients (*a*‐*l*) from four BMI groups

	Patient #	Total DSA*	Total CRM*	Total LIH*	Actual DAP*	Predicted DAP*
BMI < 18.5 kg/m^2^	Patient *a*	23.8	5.9	0.6	45.2	41.3
	Patient *b*	16.6	6.9	0.4	33.5	31.4
	Patient *c*	23.0	6.2	0.2	35.0	36.4
18.5 ≤ BMI <24.9 kg/m^2^	Patient *d*	59.6	14.0	0.9	93.5	96.4
	Patient *e*	26.0	6.0	0.4	40.2	42.0
	Patient *f*	24.6	6.8	0.3	40.3	40.0
25 ≤ BMI ≤29.9 kg/m^2^	Patient *g*	30.2	9.0	0.3	44.7	49.1
	Patient *h*	32.3	8.0	0.3	49.0	50.6
	Patient *i*	26.6	9.6	0.8	46.8	50.5
BMI ≥ 30 kg/m^2^	Patient *j*	59.6	14.0	0.9	93.5	96.4
	Patient *k*	48.2	11.5	0.6	74.8	76.8
	Patient *l*	48.6	12.5	0.5	74.0	77.2

Note:

This table demonstrates the actual dose measurements from each imaging mode and the actual DAP for a separate group of 12 UAE patients (*a*‐*l*) from four different BMI groups. BMI < 18.5 kg/m^2^, 18.5 ≤ BMI ≤24.9 kg/m^2^, 25 ≤ BMI ≤29.9 kg/m^2^ and BMI ≥ 30 kg/m^2^ groups were considered underweight, normal weight, overweight and obese respectively. The regression equation was used to calculate the predicted DAP with a high predictive capacity of 91.1‐99.3%. (UAE = uterine artery embolisation, BMI = body mass index, DSA = digital subtraction angiography, CRM = conventional roadmap, LIH = last‐image hold, DAP = Dose Area Product).

* All dose quantities are measured in Gy·cm^2^.

## Discussion

In this study, multiple linear regression analysis identified total DSA, total CRM and total LIH as the three independent predictors of radiation dose for female patients undergoing UAE. The findings demonstrated a regression model which entered and identified key predictors of dose with direct clinical applications for optimising UAE radiation dose through the control of these dose predictors. Our preliminary validation of these results across different BMI groups showed that the regression model has defined these three dose predictors to have a significant impact on the DAP exposure outcome. Further validation is required to account for diverse patients, clinical variability and procedural degrees of difficulty. Intra‐procedural adjustment of these three predictor variables can reduce the overall DAP, particularly for total DSA which contributes the highest radiation dose per imaging mode. The study outcomes can be used in a CQI programme to control and optimise radiation dose in future UAEs and other similar interventional radiology procedures.

Total DSA, total CRM and total LIH fit the model and directly correlate with DAP since all three variables are controllable and highly operator‐dependent during the procedure. The uneven distribution of data for BMI, total number of fibroids, total fibroid volume and total uterus volume were not significant as these variables are prescribed values for the presenting patient which cannot be modified or controlled prior to the procedure. DSA routinely contributes to most of the radiation dose with the aortogram and selective bilateral uterine angiograms in our UAE protocol. Any additional DSA and CRM acquisition of the internal iliac artery and/or uterine artery origins due to vascular complexity would inherently increase the total DSA and total CRM measurements from the DAP.

There has been no known research into the identification of predictors of dose for UAE. Application of our research findings to the clinical setting is important to improve the practice of interventional radiologists and radiographers when performing procedures to limit patient exposure to ionising radiation. This also adheres to the ALARA principle and promotes the safe use of radiation dose especially for our mainly reproductive‐age UAE patients. Kohlbrenner et al stated that the radiation dose during UAE should be optimised as high doses can result in skin burns and potentially increase the patient’s long‐term risk of developing cancer.^20^ Since the radiation dose is focused on the pelvis and reproductive organs, Thomaere et al measured the effective organ dose to the ovaries and uterus for forty‐one UAE patients.^21^ Despite recording an approximate mean ovarian and uterine dose of 0.04 Gy, these radiosensitive organs are vulnerable to any level of ionising radiation exposure.^21^ Therefore, the knowledge and control of dose predictors could reduce the risks of tissue effects and/or stochastic effects to this specific patient population.

From our findings, DSA was identified as the primary predictor of dose and contributed the most amount of radiation dose per imaging mode. Studies have demonstrated how DSA can be regulated to reduce patient exposure.^22^, ^23^, ^24^ White et al used a UAE protocol that acquired uterine arteriography at only 1 frame every 2 seconds and 1 fps for the aortogram, which is lower than our DSA frame rates as described in the methods.^22^ It was suggested that lowering the number of angiographic images for DSA and the frame rate can further reduce dose.^22^ Studies have reported that employing left anterior oblique (LAO) and right anterior oblique (RAO) projections can increase the radiation dose by up to 30%,^23^, ^24^ but may affect fluoroscopy time and dose when accessing the LUA and RUA origins in the PA projection. The radiation dose output would further increase exponentially if DSA imaging is used in conjunction with oblique projections and magnification. DSA may provide a high‐resolution roadmap for navigating devices through difficult vasculature and challenging uterine artery origins but this accounts for a higher radiation dose compared to CRM, LIH and fluoroscopy. According to our findings, limiting the use of DSA with lower frame rates, exposure times, magnification and obliquity can potentially further reduce the radiation dose to UAE patients.

CRM and LIH were used at the discretion of the operators and are controllable variables that the interventional radiologist and radiographers can use over DSA as a roadmap with reduced patient radiation dose.^25^, ^26^ Both of these imaging modes were used in practice with oblique projections and magnification for ipsilateral and contralateral uterine artery access. Vetter et al defined dose optimisation strategies that avoided oblique projections and used LIH instead of DSA which markedly reduced the total DAP by five times. For their 21 patients where DSA and oblique projections were used the mean DAP was 69 Gy·cm^2^, and for 25 patients where only LIH and no obliques were used the mean DAP was reduced to 13 Gy·cm^2^.^13^ LIH provides adequate diagnostic information without compromising treatment. Our regression model shows that the preferential use of the weak predictors CRM and LIH for routine roadmapping would result in a reduction in the cumulative DAP measurement.

The outcomes of this study can be integrated into a quality improvement programme to proactively monitor for changes in the outcome of the imaging process for UAE procedures. Studies in other areas of radiology have shown the effective implementation of dose optimisation methods within a CQI project to reduce cumulative radiation exposure in medical imaging.^27^, ^28^ Our interventional radiologist and radiographers can apply this model to control the identified dose predictor variables and potentially reduce our median DAP and median AK values below the recommended 100 Gy·cm^2^ and 2 Gy for radiation skin‐absorbed dose respectively.^29^, ^30^ The following angiographic techniques can further optimise dose during the UAE: active tight collimation, minimal magnification, intermittent fluoroscopy, limiting DSA exposure time and reduced object to image receptor distance (OID) to minimise pulse rate to the lowest practical level.^25^, ^26^, ^31^, ^32^ Nikolic et al reported an AK of 1.6 Gy and concluded that the exposure was unlikely to cause acute or long‐term radiation‐induced injury or pose a risk to progeny.^10^ At our institution a longitudinal study can validate the effects of irradiating the pelvic region, while the application of the dose predictors can improve the quality of controlling radiation dose for our future UAE patients with adherence to the ALARA principle.

This study had certain limitations. There are a single‐centre and single‐operator bias inherent within the methodology. However, a single operator does strongly favour towards standardised operator variability, and a large diverse patient cohort has been employed to minimise any patient bias. The regression model has potential to predict actual dosages received based on several imaging input parameters. This would be verified by a multi‐centre study in the Australian or international context to reflect broader operator experience and further test and validate the regression equation. The model can be limited in its use to attain dose optimisation as some factors that contribute to the DAP are not controllable by the operators, are intrinsic to the imaging system and defined by the imaging demanded by the clinical circumstance. Another limitation was that other parameters and radiation dose measurements due to magnification, obliquity and number of DSA runs were not included in the data collection.

Future studies are required for establishing a potential prediction model using our recently upgraded angiography unit with dose‐limiting technology and auxiliary software, where predictors of dose may change and be considered machine‐dependent. Following derivation and validation, the model can be used as a method of quality improvement to quantify individual contributions of various dose predictor variables.^6^

In conclusion, this study identified key imaging predictors of dose which can be controlled and optimised during UAE for this mainly reproductive‐age patient demographic. All clinicians can utilise the knowledge from our regression model to optimise the dose predictors in conjunction with dose‐limiting angiographic techniques and the high volume experience of UAE procedures, thereby reducing the cumulative DAP outcome. This implementation further reduces the risks from ionising radiation exposure and has substantial implications to improve radiation safety and the quality of practice when performing UAE or other similar interventional radiology procedures.

## Conflict of Interest

The authors declare no conflict of interest.
